# Ducklings Were Susceptible to Swine Acute Diarrhea Syndrome Coronavirus Under Experimental Conditions

**DOI:** 10.3390/microorganisms13092122

**Published:** 2025-09-11

**Authors:** Teng Zhang, Longfa Li, Jiayi Wang, Jiale Yao, Guoqing Xu, Chaoliang Leng, Yong Wang, Lunguang Yao

**Affiliations:** 1 Henan Provincial Engineering and Technology Center of Health Products for Livestock and Poultry, School of Life Science, Nanyang Normal University, Nanyang 473000, China; 20211039@nynu.edu.cn (T.Z.); 2024086001036@nynu.edu.cn (L.L.); 2024086004002@nynu.edu.cn (J.W.); 2022086001028@nynu.edu.cn (J.Y.); lenghan1223@126.com (C.L.); 2 College of Veterinary Medicine, Anhui Agricultural University, Hefei 230036, China; xu2734486609@163.com

**Keywords:** severe acute diarrhea syndrome coronavirus, susceptibility, duckling, cross-species infection

## Abstract

Swine acute diarrhea syndrome coronavirus (SADS-CoV), similar to other coronaviruses, exhibits extensive host tropism and has caused huge losses to the pig industry since its first outbreak in 2017. However, the susceptibility of SADS-CoV in waterfowl remains unclear. In the present study, 10-day-old ducklings were orally administered 5.95 log_10_ TCID_50_ (the tissue culture infective dose 50%) of SADS-CoV, with a medium serving as a control treatment, to assess ducklings’ susceptibility. Results indicated that the ducklings exhibited mild diarrhea symptoms, experienced slow weight gain, and one duckling died seven days after inoculation. Histopathological examination revealed that the viral infection caused pathological damage to the spleen, intestine, and lungs. Tissue immunofluorescence demonstrated viral replication in the spleen, lungs, and intestine. This study provides the first evidence that SADS-CoV can infect ducklings under laboratory conditions. Given that waterfowl may serve as significant reservoirs for various viruses, this finding raises considerable concerns.

## 1. Introduction

The outbreak of severe acute respiratory syndrome coronavirus 2 (SARS-CoV-2) in 2019 has resulted in significant human mortality and substantial economic losses worldwide, highlighting the inadequacy of our understanding of coronaviruses [1,2]. Cross-species transmission is a critical characteristic of coronaviruses and serves as a primary mechanism by which they threaten human health [3,4]. Therefore, a comprehensive understanding of the host infection range of coronaviruses is essential for effective detection, early warning, and prevention strategies.

The first outbreak of swine acute diarrhea syndrome coronavirus (SADS-CoV) occurred in 2017. SADS-CoV is a member of the genus *Alphacoronavirus* and subgenus *Rhinacovirus* [5,6,7]. SADS-CoV is an enveloped, single-stranded, positive-sense RNA virus. Since its emergence, SADS-CoV has rapidly spread to Guangdong, Guangxi, Jiangxi, and Fujian provinces in China [8,9,10]. By 2023, an additional outbreak of SADS-CoV was reported in central China, indicating significant potential for widespread transmission throughout the country [7]. Even worse, a retrospective study in 2025 found that the SADS-CoV virus had already spread to Vietnam [11]. Meanwhile, a serological survey indicates that SADS-CoV has already been prevalent in multiple provinces of Korea, although the prevalence rate is relatively low (8.7%) [12]. These suggest that SADS-CoV has spilled over and may be widely prevalent in East Asia, posing a risk of global dissemination.

SADS-CoV has garnered significant attention due to its close genetic relationship with the bat coronavirus BtRp-AphaCoVGX2017-Q225, exhibiting over 95% genome homology, which suggests a substantial potential for cross-species transmission [11,13,14]. The range of hosts that SADS-CoV can infect is under constant study and has become a focal point of research. Notably, in 2022, SADS-CoV was demonstrated to infect mice, in addition to its natural host, pigs, and was found to be lethal to newborn mice, including mouse strains BALB/c and C57BL/6J [15,16,17]. Furthermore, in the same year, it was also shown that SADS-CoV can infect chickens, leading to clinical symptoms such as respiratory difficulties and depression [18].

Considering that ducklings are significant aquatic birds, they possess a greater range of mobility and closer contact with humans compared to pigs, chickens, and mice. Therefore, identifying the susceptibility of SADS-CoV in ducklings is of the utmost importance. In the present study, we selected 10-day-old ducklings and orally administered them with a 5.95 log_10_ tissue culture infectious dose 50% (TCID_50_) of SADS-CoV to assess their susceptibility to infection by the virus. The results indicated that the infected ducklings exhibited mild diarrhea symptoms, experienced slow weight gain, and one duckling died seven days after inoculation. Histopathological and immunofluorescence examinations demonstrated viral replication in the spleen, lungs, and intestine. Our findings show that ducklings are experimentally susceptible, raising concerns about their potential roles as reservoirs that need further investigation in the field.

## 2. Materials and Methods

### 2.1. Cells and SADS-CoV Preparation

African green monkey kidney (Vero) cells were stored in our laboratory and cultured in Dulbecco’s Modified Eagle’s Medium (DMEM; Solarbio, Beijing, China) containing 10% fetal bovine serum and 1% penicillin-streptomycin at 37 °C in a 5% CO_2_ atmosphere. SADS-CoV/HNNY/2023 (GeneBank Accession number: PP069800) was isolated in our laboratory and proved to be a highly pathogenic strain by animal experiments.

### 2.2. Virus Titer Determination

The tissue culture infective dose 50% (TCID50) of SADS-CoV was determined using Vero cells according to a previously reported method [18]. In brief: Vero cells were seeded in 96-well plates two days in advance, and on day three when the cell count was above 90%, the cells were washed three times with PBS (Phosphate-Buffered Saline) to remove fetal bovine serum. Ten-fold serial dilutions of SADS-CoV were then added to the cells at trypsin concentrations of 10 μg/mL in eight replicates per gradient. After 48 h, the viral cytopathic effect (CPE) was determined by the immunoperoxidase monolayer assay (IPMA). Finally, the TCID_50_ of SADS-CoV was calculated according to the Reed–Muench method.

### 2.3. Animal Experiment Design

Twenty 8-day-old cherry valley ducklings were purchased from a hatchery in Nanyang, Henan. The ducklings were judged by experienced veterinarians to be healthy, in good mental condition, with no diarrhea, a normal diet and water intake, and without any damage to appearance when purchased. After arriving at the animal room, the ducklings were fed with commercialized feed and purified water. And the ducklings were initially free of SADS-CoV which was confirmed by qPCR using stool samples and oral swab samples. The ducklings were randomly divided into two groups (n = 10) and placed in separate rooms, respectively. After two days of acclimation, Group I were orally infected with 5.95 log_10_ TCID_50_ SADS-CoV/HNNY/2023 strains. And Group II were orally administered an equal volume of DMEM and served as the control.

Clinical signs, weight, and rectal temperatures were measured daily until 10 dpc (days post challenge). At 10 dpc, the surviving ducklings were humanely euthanized using carbon dioxide and the typical lesions in the spleen, intestine (jejunum), and lungs were collected and fixed with paraformaldehyde for 24 h. Then, tissues were sectioned to 3–4 μm slices and one part was stained with hematoxylin and eosin (H&E) for histopathological analysis. On another part, tissue immunofluorescence analysis was performed using mAbs (monoclonal antibody) anti-SADS-CoV-N protein by Servicebio (Wuhan, China). In addition, diarrhea severity was scored using the following criteria: 0 = normal; 1 = soft (cowpie); 2 = liquid with some solid content; 3 = watery with no solid content; and 4 = death [19].

### 2.4. SYBR Green qPCR

The viral genome was extracted using TaKaRa MiniBEST Viral RNA/DNA Extraction Kit Ver.5.0 (TaKaRa, Dalian, China), and reverse transcription was performed using the All-in-One Script RTpremix (Kermey Biotech, Zhengzhou, China). The primers encoding the N protein gene were as follows: SADS-CoV-N-qF: 5′-ATTACCACCAGACCTGACT-3′; and SADS-CoV-N-qR: 5′-TGATTGCGAGAACGAGAC-3′. The amplified fragment was 75 base pairs. The plasmid pET28a-SADS-CoV-N was constructed, sequenced by Sangon Biotech, and used as the standard plasmid to calculate the number of copies.

### 2.5. Detection of Serum IgG Antibodies Against SADS-CoV

SADS-CoV-specific antibodies were detected by ELISA (Enzyme-Linked Immunosorbent Assay) based on the whole virus as described previously [18]. In brief, viral particles were purified by sucrose density gradient centrifugation and the protein concentration was determined using the BCA method (Thermo Fisher, Waltham, MA, USA). The coating concentration of the antigen was determined at 10 ng/well by checkerboard method. Serum was diluted at 1:100 and then used for detection. The secondary antibody was an HRP-conjugated rabbit anti-duck IgY (Biodragon, Suzhou, China) at 1:5000 dilution. After obtaining the OD_450_ value, 2.1 times the average value of the negative serum was taken as the positive cutoff value.

### 2.6. Ethics Approval

The animal experiments were performed in strict accordance with the guidelines of the Animal Care and Use Committee of Nanyang Normal University, which functions to ensure the ethical and humane treatment of experimental animals (approval number: 2025NYNU012).

## 3. Results

**Figure 1:**
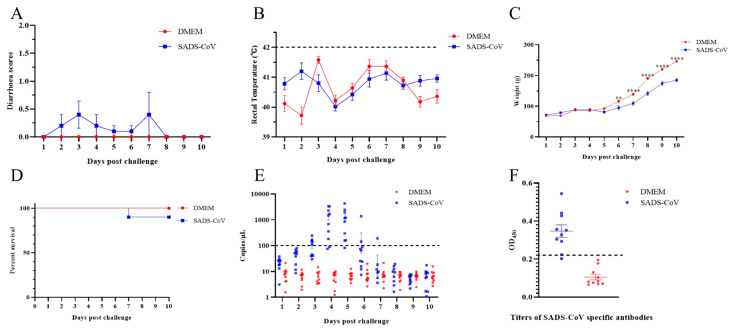
Overall clinical symptoms of ducklings infected with SADS-CoV. The 10 day-old ducklings were randomly divided into two groups (n = 10), and one group (SADS-CoV) were orally infected with 5.95 log_10_ TCID_50_ SADS-CoV/HNNY/2023 strains per duckling. The other group (DMEM) was orally inoculated with the same volume of DMEM in the same manner as the control. (**A**) Daily clinical symptoms were scored using the following criteria: 0 = normal, 1 = soft (cowpie), 2 = liquid with some solid content, 3 = watery with no solid content, and 4 = death. The rectal temperature (**B**) and weight of the ducklings (**C**) were measured daily. The dashed line represents the reference value for fever in ducklings (42 °C). The data were analyzed by two-way ANOVA using GraphPad Prism software 8.0.2 (** *p*-value < 0.01, and **** *p*-value < 0.0001) with a 95% confidence interval. (**D**) The survival curve after viral challenge was determined. (**E**) The viral loads of daily anal swabs were determined by quantitative PCR. The dash line represents the detection limit of qPCR. (**F**) Detection of SADS-CoV-specific antibody titers in serum. The dashed line represents the positive cutoff value (2.1 times the average value of the negative serum).

Considering that SADS-CoV has been shown to infect chickens, we aimed to identify the susceptibility of ducklings to SADS-CoV, as a species of waterfowl with closer contact opportunities with pigs and humans. The 10-day-old ducklings (n = 10) were injected orally with 5.95 log_10_ TCID_50_ SADS-CoV. Results showed that two ducklings developed diarrhea at 2 dpc, four ducklings developed diarrhea at 3 dpc, and two ducklings developed diarrhea at 4 dpc in the SADS-CoV group. All the ducklings stopped diarrhea on day 5, except for one duckling with continued diarrhea until death on day 7 (Figure 1A). The relatively mild clinical symptoms suggest that the majority of infections may be subclinical. We also monitored the rectal temperature changes throughout the experiment and, as in pigs, the SADS-CoV infection did not cause an increase in body temperature (Figure 1B). Given that the SADS-CoV infection severely reduced weight gain in piglets, we also monitored weight gain in ducks. The results showed that the weight gain of the infected ducklings decreased significantly from the 6 dpc to the end of the experiment (Figure 1C). In addition, the death of a duckling appeared to indicate the potential pathogenicity of SADS-CoV for ducklings (Figure 1D). None of the ducklings orally infected with DMEM experienced diarrhea or death during the whole experiment.

To monitor virus shedding, daily anal and oral swabs were collected and then the virus genome was extracted and detected via qRT-PCR. The results showed that the virus on anal swabs of the infected ducklings was first observed at 3 dpc and peaked at 4 and 5 dpc (Figure 1E). No viral shedding was detected in oral swabs. In addition, ELISA results showed that at the end of the experiment, 8/10 ducklings developed SADS-CoV-specific antibodies (Figure 1F).

**Figure 2:**
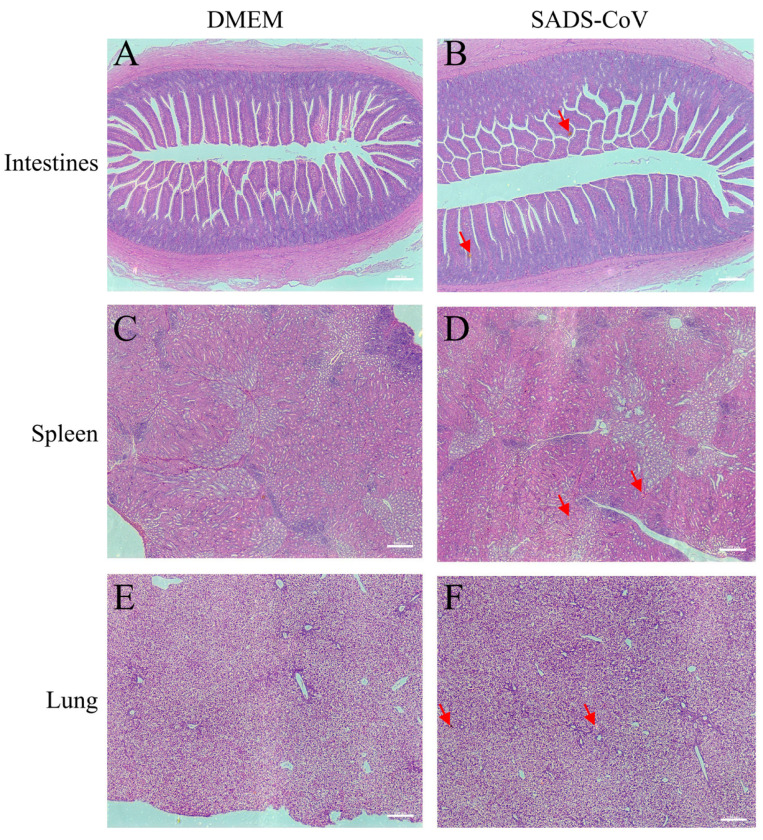
Histopathology of representative organs from the challenged ducklings and the control. Intestines (jejunum) tissues (**A**,**B**), spleen tissues (**C**,**D**), and lungs ((**E**,**F**), at the bottom) were harvested at 10 dpc. Tissues were sectioned to 3–4 μm slices and stained with H&E and analyzed at 20× magnification. The red arrows indicated a portion of the pathological injuries such as inflammation or hemorrhage. Scale bar: 100 μm. DMEM: Dulbecco’s Modified Eagle’s Medium, SADS-CoV: swine acute diarrhea syndrome coronavirus.

**Figure 3:**
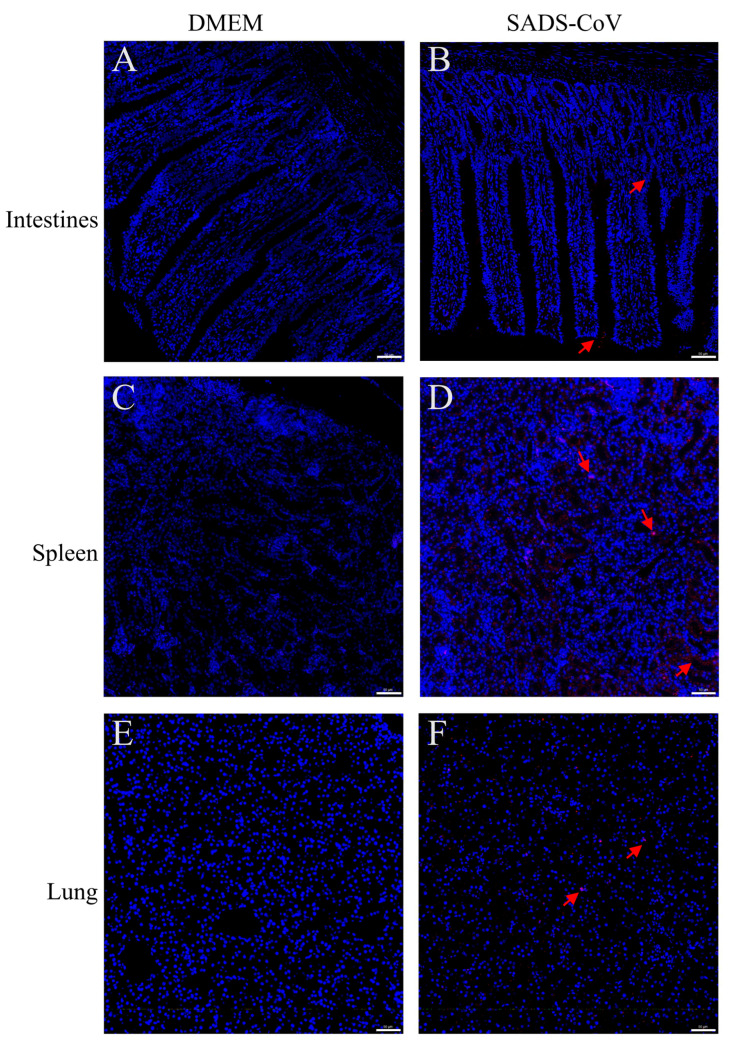
Immunofluorescence histochemistry examination of representative organs from challenged ducklings and control. Intestines (Jejunum) tissues (**A**,**B**), spleen tissues (**C**,**D**), and lungs ((**E**,**F**), at the bottom) were harvested at 10 dpc. Tissues were sectioned to 3–4 μm slices and immunofluorescence examination was performed using an mAb specific for SADS-CoV-N protein. Scale bar: 50 μm. The red arrows indicated the a portion of the SADS-CoV antigen. DMEM: Dulbecco’s Modified Eagle’s Medium, SADS-CoV: swine acute diarrhea syndrome coronavirus.

To further examine whether SADS-CoV infection causes pathological damage to tissues and organs, we collected typical sites of the lungs, spleen, and intestine for histopathological examination. During the dissection process, the intestinal mucosa of the infected ducklings exhibited slightly reddish coloration due to mild congestion. In addition, the intestinal contents contained purulent secretions, with no significant changes in the thickness of the intestinal wall. Combined with the subsequent histopathological findings of only mild congestion and edema in the intestinal mucosa under the microscope, and the essentially intact villous structure, the results showed that SADS-CoV infection did not cause the intestinal villi of the ducklings to fall off, but it introduced a small amount of inflammation and bleeding (Figure 2A,B). In addition, SADS-CoV infection also caused hemorrhages and neutrophil infiltration in the spleen and lungs (Figure 2C–F). To further detect the viral load in tissues, immunofluorescence detection was performed. Results showed SADS-CoV can proliferate in the intestine, spleen, and lungs, but the spleen had a higher viral load (Figure 3A–F). Although the data in the present study demonstrated that the ducklings were susceptible to SADS-CoV under laboratory conditions, there is no solid evidence of natural infection or effective transmission.

## 4. Discussion

SADS-CoV poses a significant threat to global swine production, causing severe acute diarrhea, high mortality in piglets, and substantial economic losses since its emergence in 2017 [15,20]. Its broad host tropism, previously documented in mammals, now extends to avian species [18]. In this study, we provide the first experimental demonstration that SADS-CoV can infect and replicate in ducklings under laboratory conditions. The successful oral infection, confirmed by viral replication detected via immunofluorescence in key tissues (spleen, lungs, intestine), established ducklings as a susceptible host species for this primarily porcine pathogen. This finding significantly expands the known host range of SADS-CoV and beyond.

Infection by SADS-CoV resulted in observable clinical disease in ducklings, characterized by mild diarrhea and, crucially, impaired weight gain. Histopathological examination revealed tissue damage in the spleen, intestine, and lungs. Immunofluorescence assays demonstrated viral proliferation in the lungs, spleen, and intestines, with the highest viral load observed in the spleen, which was inconsistent with the highest viral load in the midbrain of mice and in the upper intestine of piglets, which may be a result of species differences [7,16]. Although the clinical symptoms in ducklings appeared milder than the severe, often fatal disease observed in piglets, the pathological effects confirm that SADS-CoV was not merely present but actively causes disease in ducklings. The relatively mild clinical symptoms suggest that the majority of infections may be subclinical. During the animal experiments, we strictly controlled the environmental conditions such as temperature, humidity, water supply, and feed. No environmental stressors such as water shortage, temperature differences, or overcrowding that could cause individual differences were observed. More importantly, the tissue immunofluorescence results of the deceased duckling showed significant viral replication in the intestinal tissues (Figure 3), and HE staining confirmed the presence of minor inflammatory damage in the intestines. These results were more inclined to reflect that the virus replicated within the individual and triggered a relatively obvious local immune response, which may be related to the death outcome. The mortality of one duckling further underscores the pathogenic potential of the SADS-CoV for ducklings. In addition, the negative oral swab but positive anal swab may indicate different tropisms of the SADS-CoV infection. For piglets, SADS-CoV infection primarily affects the intestines. For chickens, SADS-CoV can be shed through both throat swabs and anal swabs, mainly causing respiratory diseases [18]. For ducklings, no significant virus shedding through throat swabs has been observed, and consequently, no significant respiratory symptoms have been noted. SADS-CoV infection in the hosts (ducklings and pigs) did not cause fever, which may represent the viral active suppression of the host’s fever response. Low-dose infections may be asymptomatic or subclinical, making clinical observation insufficient for early outbreak detection. Our study emphasizes the necessity of active viral surveillance for SADS-CoV.

Recent studies show that other porcine coronaviruses, such as PDCoV, could also infect ducklings and cause symptoms such as diarrhea [21], indicating that the SADS-CoV infection of ducklings may not be a simple isolated event. Considering the complexity of avian coronaviruses, there may be significant biosecurity risks if they co-infect the same duckling with swine coronaviruses. Therefore, it is extremely important to detect the prevalence of coronavirus in duckling populations.

Birds, particularly waterfowl, serve as significant natural reservoirs and amplification hosts for various viruses, including the avian influenza virus, Newcastle disease virus, West Nile virus, and Tembusu virus [22,23,24,25]. The demonstration of SADS-CoV infection in an avian species in the present study highlights the virus’s broad host tropism and adaptive potential. This cross-species infectivity needs heightened vigilance. There is an urgent need for field surveillance to ascertain whether natural SADS-CoV infections occur in wild and domestic waterfowl populations. Additionally, research is essential to evaluate the risk that ducklings pose in transmitting the virus to pigs or other livestock under natural conditions and to comprehend the potential for viral evolution within a new avian host. These findings underscore the importance of monitoring and controlling SADS-CoV.

## Data Availability

The original contributions presented in this study are included in the article. Further inquiries can be directed to the corresponding authors.
